# Minimally invasive breast cancer excision using the breast lesion excision system under ultrasound guidance

**DOI:** 10.1007/s10549-020-05814-z

**Published:** 2020-08-01

**Authors:** W. B. G. Sanderink, L. J. A. Strobbe, P. Bult, M. S. Schlooz-Vries, S. Lardenoije, D. J. Venderink, I. Sechopoulos, N. Karssemeijer, W. Vreuls, R. M. Mann

**Affiliations:** 1 Department of Medical Imaging/Radiology, Radboud University Medical Center, Geert Grooteplein 10, 6525 GA Nijmegen, The Netherlands; 2 Department of Surgical Oncology, Canisius-Wilhelmina Hospital, Nijmegen, The Netherlands; 3 Department of Pathology, Radboud University Medical Center, Nijmegen, The Netherlands; 4 Department of Surgery, Radboud University Medical Center, Nijmegen, The Netherlands; 5 Department of Radiology, Canisius-Wilhelmina Hospital, Nijmegen, The Netherlands; 6 Department of Pathology, Canisius-Wilhelmina Hospital, Nijmegen, The Netherlands

**Keywords:** Breast, Biopsy, Breast cancer, Minimally invasive, Vacuum

## Abstract

**Purpose:**

To assess the feasibility of completely excising small breast cancers using the automated, image-guided, single-pass radiofrequency-based breast lesion excision system (BLES) under ultrasound (US) guidance.

**Methods:**

From February 2018 to July 2019, 22 patients diagnosed with invasive carcinomas ≤ 15 mm at US and mammography were enrolled in this prospective, multi-center, ethics board-approved study. Patients underwent breast MRI to verify lesion size. BLES-based excision and surgery were performed during the same procedure. Histopathology findings from the BLES procedure and surgery were compared, and total excision findings were assessed.

**Results:**

Of the 22 patients, ten were excluded due to the lesion being > 15 mm and/or being multifocal at MRI, and one due to scheduling issues. The remaining 11 patients underwent BLES excision. Mean diameter of excised lesions at MRI was 11.8 mm (range 8.0–13.9 mm). BLES revealed ten (90.9%) invasive carcinomas of no special type, and one (9.1%) invasive lobular carcinoma. Histopathological results were identical for the needle biopsy, BLES, and surgical specimens for all lesions. None of the BLES excisions were adequate. Margins were usually compromised on both sides of the specimen, indicating that the excised volume was too small. Margin assessment was good for all BLES specimens. One technical complication occurred (retrieval of an empty BLES basket, specimen retrieved during subsequent surgery).

**Conclusions:**

BLES allows accurate diagnosis of small invasive breast carcinomas. However, BLES cannot be considered as a therapeutic device for small invasive breast carcinomas due to not achieving adequate excision.

## Introduction

Due to a substantial portion of breast cancers being detected at screening, the average size of newly detected breast cancers is decreasing, with 53% of them being below 2 cm [1]. For small cancers, breast conserving therapy (BCT), including wide local excision and radiation therapy, has largely replaced mastectomy [2]. The trend towards BCT has been set despite clear evidence that local surgical excision alone frequently leaves residual cancer deposits in the breast [3, 4]. However, since the addition of radiation therapy decreases the local recurrence risk, BCT is as safe as mastectomy [5, 6]. Nonetheless, residual cancer in the resection margin is predictive for recurrence, which results in poorer overall survival [6]. Consequently, assessment of tumor involvement of the surgical resection margin has become standard of care [7].

Recently the breast lesion excision system (BLES™, Medtronic Inc., Dublin, Ireland) has been introduced for breast cancer diagnosis [8]. Briefly, the device, designed for diagnostic breast biopsy, excises a lump of tissue through a very small skin incision under mammographic or ultrasound (US) guidance. The size of the extracted lump is dependent on the biopsy needle chosen, which is available in diameters of 12 mm, 15 mm, and 20 mm [9].

There are a few reports suggesting that, in a diagnostic setting, up to 66% of invasive cancers are completely excised using the BLES, albeit these studies did not aim to excise the entire lesion, and where mainly performed under mammographic guidance [9–16].

Very little literature exists on the use of BLES under US guidance [17, 18], with only Niinikoski et al. [18] reporting a complete excision rate under US guidance of 46.6%. US guidance allows for real-time feedback of the needle position during biopsy, and has been shown to be beneficial for surgical tumor excision [19, 20]. Therefore, US seems to be a logical choice as the guidance technique when BLES is used as a therapeutic device.

Therefore, the aim of this study was to evaluate whether it is feasible to excise small breast cancers completely using the BLES system under US guidance.

## Materials and methods

### Study design and patient population

This prospective multi-center study was approved by the local ethical review board, and all study participants provided written informed consent. Two different hospitals situated in Nijmegen, the Netherlands, were participating (Radboud University Medical Center, an academical hospital and Canisius-Wilhelmina Hospital, a district hospital). Patients with histologically proven invasive breast cancer based upon a diagnostic 14G core needle biopsy, and with a maximum diameter of 15 mm as assessed at US and mammography were included in our study. The tumor had to be clearly visible with US according to the radiologist who performed the primary evaluation. Patients with an indication of more extensive disease on imaging (e.g., an area of calcifications adjacent to the mass) were excluded. Pregnant patients, patients with breast implants, and patients with implanted electronics, such as a cardiac pacemaker, were not suitable to undergo the BLES biopsy and therefore also excluded. Furthermore, patients were excluded when the breast lesion was situated closer than 6 mm to the dermis, nipple, or pectoral muscle.

### Imaging

In all subjects the lesion diameter and the absence of a multifocal tumor was verified with magnetic resonance imaging (MRI). For this, all patients were scanned on a 3T system with a 16 channel breast coil (Skyra, Siemens, Erlangen, Germany), using a state-of-the-art full diagnostic protocol as previously described by Dalmis et al. [21], including high resolution T1 weighted pre- and post-contrast acquisitions. Tumor diameter was assessed in three orthogonal directions, on both the original images obtained two minutes after contrast administration and on the subtracted images generated from pre- and post-contrast acquisitions by one of two breast radiologists with 12 and 16 years of experience. When the maximum lesion diameter was confirmed as being ≤ 15 mm at MRI, the subjects could continue in the study.

### BLES and surgical procedure

The BLES procedure was scheduled directly preceding the regularly planned surgery, to take place when the patient was already under general anesthesia, to minimize the burden of the study on the patient.

Although the patient positioning was optimized for surgery, when required the table could be tilted to improve the accessibility of lesions for the BLES procedure. All BLES procedures were performed by one of two radiologists using a 20 mm disposable BLES needle under US guidance using an US system equipped with a 34 mm 4–12 MHz linear probe (L12-4 Broadband linear array transducer, Philips, Eindhoven, the Netherlands). The 20 mm probe used during this study enables an excision of a spheroid specimen with a maximum thickness of 20 mm.

Following the BLES procedure, the BLES excision cavity and at least 1 cm of surrounding tissue was excised by one of two dedicated breast surgeons with 22 and 26 years of experience. Both specimens (BLES and surgical) were marked with sutures by the surgeon to document the orientation according to standard protocols and sent to histopathology.

### Histopathology analysis

The specimens were processed as per standard procedures at the pathology department, including X-ray imaging of both specimens (intact and sliced). The specimens were inked on the external surfaces and sliced perpendicular to the longest axis of the specimens. These slices were serially embedded and examined using standard pathological analysis (hematoxylin and eosin staining) as well as advanced pathological evaluation such as immunohistochemistry. Margin assessment was performed separately for the BLES excision and the surrounding surgical specimen by one of two breast pathologists with 10 and 25 years of experience. The residual tumor burden and histology in the surgical specimen was also assessed. In accordance with the Dutch Breast Cancer Guidelines, *adequate excision* was defined as having no more than focal involvement of the resection margins, which is defined as foci of invasive tumor and/or adjacent ductal carcinoma in situ (DCIS) touching four mm or less of the inked margin [22].

For each lesion, we assessed concordance between the histopathological diagnosis obtained at diagnostic biopsy [core needle biopsy (CNB), BLES, and surgical excision].

### Follow-up

All patients had a post procedure follow-up appointment within two weeks after surgery to discuss pathology results and examine the healing process of the incisions and, if present, deal with any complications such as hematoma or infection.

### Data analysis

The mean values and the respective standard deviations were used to describe continuous measurements, such as the diameter and margins in mm, while frequencies and percentages were used for categorical variables such as the lesion types, complications, and concordance between BLES and surgery.

All statistical analyses were performed using SPSS software version 25 (SPSS Inc., Chicago, IL, USA).

## Results

From February 2018 to July 2019, a total of 22 patients who had histologically confirmed invasive carcinomas with a diameter ≤ 15 mm on US and mammography were enrolled in the study. Eleven patients (50%) were subsequently excluded due to MRI findings (*n* = 10) or because the BLES was not available at the time of surgery (*n* = 1) (Table 1). Characteristics of patients and lesions are detailed in Table 2.

**Table 1:** Reason for subsequent exclusion from BLES procedure

	*N* = 11
BLES not available	1
MRI findings	10
>15 mm	3
Multifocal	1
>15 mm and multifocal	6

**Table 2:** Baseline characteristics of patients and lesions treated with the BLES

	Median (range)
Age	60 years (5173 years)
Lesion diameter	
US	8 mm (6–11 mm)
MRI	11.8 mm (8–13.9 mm)
BLES specimen	
Length	18.9 mm (12–25 mm)
Thickness	8.8 mm (6–12 mm)
	*n* (%)
BI-RADS	
4	1 (9.1%)
5	10 (90.9%)
Radiological findings	
Round mass	1 (9.1%)
Oval mass	2 (18.2%)
Irregular mass	8 (72.7%)
ACR density classification	
A (Almost entirely fatty)	1 (9.1%)
B (Scattered fibroglandular density)	8 (72.7%)
C (Heterogeneously dense)	2 (18.2%)
D (Extremely dense)	0 (0%)
Histopathological findings	
Invasive carcinomas of no special type (NST)	10 (90.9%)
Invasive lobular carcinoma	1 (9.1%)
Radiofrequency-related thermal damage	
Mild (< 0.5mm)	6 (54.5%)
Moderate (0.6–1 mm)	3 (27.3%)
Extensive (1.1–1.5 mm)	2 (18.2%)

**Fig. 1:**
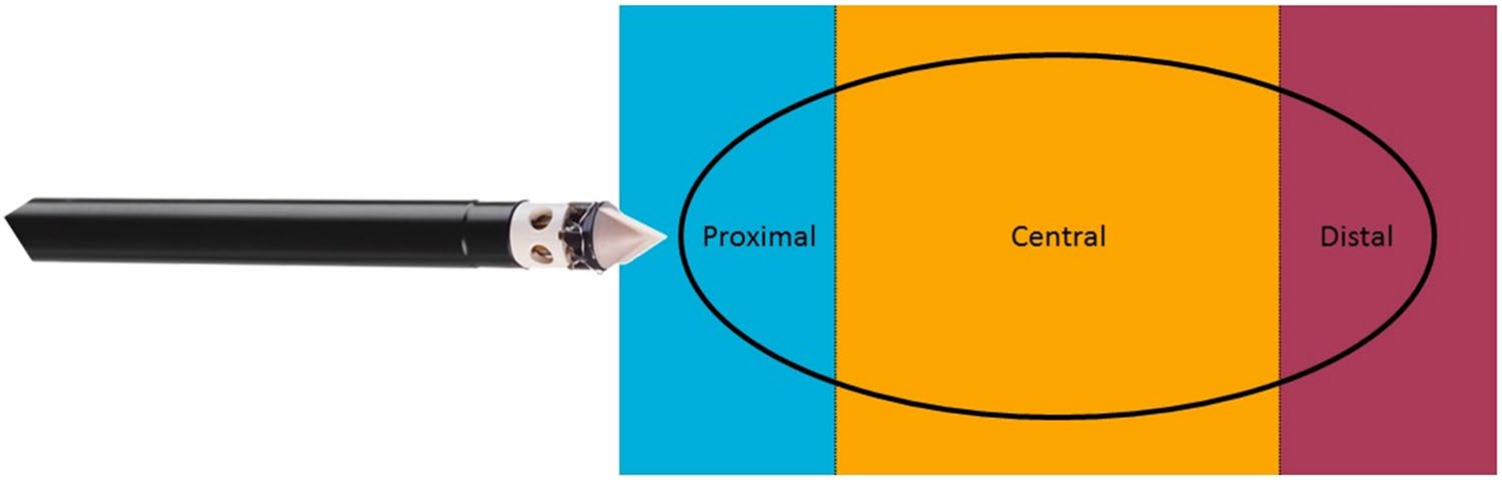
Location indication with respect to the BLES needle

At histopathological analysis of the surgical resections, the BLES biopsy cavities were identified in all cases. None of the BLES biopsies were adequate. Ten excisions (90.1%) had more than focal involvement of the resection margins and one excision (9.1%) had focal involvement with the majority of the tumor situated in the surgical specimen. Margin assessment of the BLES specimens was well possible in all cases and thermal damage had no influence on the evaluation by the pathologist. Margins were usually compromised on both sides of the specimen, indicating that the targeting was accurate, but the excised volume was too small (Fig. 1; Table 3). Residual tumor was present in all surgical excision specimens. In all cases this correlated with the positive margin seen on the BLES specimen. In the surgical resection the mean depth of the residual tumor was 3.3 mm (range: 1 –9 mm) measured perpendicular to the BLES biopsy cavity.

**Table 3:**
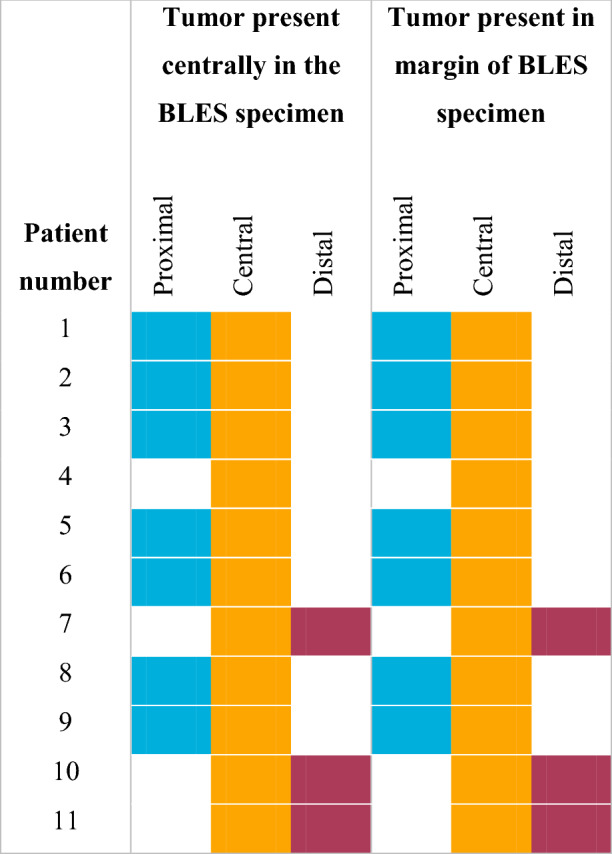
Location in BLES resection where tumor is present centrally in the BLES specimen and where margins are positive in the BLES specimen

A technical complication occurred in one case (9.1%), due to the retrieval of an empty BLES basket. However, it was possible to retrieve the BLES specimen during subsequent surgery. There were no other adverse events or post procedure complications, such as infection, hematoma, wound healing problems or unexpected scarring. Histological results were identical for CNB, BLES, and surgical specimen in all lesions.

## Discussion

Previous studies have shown that the BLES is a safe and accurate diagnostic device, and a good alternative to vacuum assisted biopsy and CNB [9, 10, 15, 16]. However, our study shows that the evaluated BLES needle (diameter of 20 mm) is too small for US guided excision of small invasive breast cancers.

Based on our results, BLES cannot be considered as a therapeutic substitute to surgical excision. In this study, we did not observe a single adequate excision of any lesion according to the Dutch Breast Cancer Guidelines (no more than focal margin involvement [22], even though lesions in this study were carefully selected through prior imaging. Previous studies have suggested that BLES could enable complete excision of small invasive lesions [9, 11, 12, 15, 23–25], with a success rate of up to 62.5%. However, most of these studies had a diagnostic focus, without aiming to excise the entire lesion, and used a different definition of an adequate BLES resection. In addition, all these studies included a time interval between the BLES procedure and the surgical re-excision, which may explain the outcome differences between them and this study. This is because, as reported by Nasir et al. [26], residual malignant cells may be eliminated during the wound healing process that occurs during the time interval between the lumpectomy and the re-excision in case of positive margins. In a similar vein, Wiley et al. [27] reported that an increased time interval between initial lumpectomy and re-excision resulted in a decreased incidence of residual disease.

It might be worth evaluating how important clear margins are for the treatment of breast cancer. The minimally accepted resection margin for breast conserving surgery above which a re-excision is advised has been already debated for years. The Society for Surgical Oncology and the American Society for Radiation Oncology (SSO-ASTRO) and the European Society for Medical Oncology (ESMO) recommend *no ink on tumor* as an adequate margin for invasive breast cancer [28, 29]. However, the Dutch Breast Cancer Guidelines considers the recurrence risk for focally (≤ 4 mm) positive margins after BCT (resection followed by radiation treatment) acceptable [22]. Vos et al. concluded that this focal involvement is usually caused by radial extensions (spicules) of the tumor or residual DCIS [30]. In another study, Vos et al. state that omitting re-excision for focally positive margins does not impair the 5-year disease-free and 10-year overall survival rates, provided that adjuvant whole-breast irradiation is given, including a boost to the tumor bed [31]. Accordingly, the treatment combination of BLES biopsy to excise the tumor bulk with subsequent adjuvant breast irradiation might be a potential option to explore in the upcoming field of minimally invasive treatment of breast cancer. Of course, oncological safety, with recurrence rates and breast cancer related mortality should be the endpoint of such studies.

All BLES procedures in our study were performed under US guidance. With US guidance there is good 3D orientation and positioning with the BLES needle, in addition to real-time imaging feedback during the procedure. However, the adequate excision rate was much lower in comparison to other studies that reported the performance of the procedure under stereotactic guidance. Milos et al. [24] and Papapanagiotou et al. [25] performed BLES resections with stereotactic guidance that resulted in a complete excision rate of 40% and 48.8%, respectively, for invasive breast lesions. The compression of the breast, which is required for stereotactic guidance, likely provides better tissue immobilization, fixing the lesion during the procedure. It can be hypothesized that this relative fixation results in fewer positive margins at the distal poles of the ellipsoidal specimen.

In our study, none of the specimen margins were free of tumor cells. This is not surprising given the fact that the specimens had an ellipsoidal shape, measuring approximately 19 mm in length and 9 mm in thickness. Therefore, it proved impossible to retrieve a specimen with a thickness of 20 mm, which we expected from a basket with dimensions of 20 mm by 25 mm. This seems to confirm the results of Christou et al. [32], who reported comparable average specimen dimensions for the 20 mm probe (20 mm in length and 10 mm in thickness). Killebrew et al. [16], did, however, report larger dimensions for the smaller 15 mm probe (21 mm in length and 15 mm in thickness). To excise even very small lesions completely it is thus necessary that this lesion is perfectly centered in the specimen, which is quite challenging under US guidance. After starting the deployment of the basket, it is not possible to re-adjust the needle location using the available real-time imaging feedback, even if the deployment itself may cause movement of the lesion due to mechanical effect on the tissue, which may push the target aside during the excision. In this study, tumor cells were observed in the margins at the poles of the ellipsoid (see Fig. 1; Table 3). In initial procedures it was mainly observed involvement of the proximal pole. This implies that the needle tip was positioned too close to the lesion, and therefore the basket was not opened wide enough when reaching the lesion. However, positioning the needle tip a little further away from the lesion resulted in involvement of the distal pole, due to too early closure of the basket. In all patients the margin was involved centrally, mainly due to the fact that the lesions were wider than the maximum thickness of the specimens. Therefore, it seems unfeasible to excise lesions completely with free margins with the currently available probes, even for very carefully selected small invasive malignancies.

Based on our results and previous studies, we would advise to consider the BLES as a therapeutic device only in specific situations and for specific lesions. First, the BLES may be a good alternative for surgical excision of small benign lesions [11, 18, 33]. However, in recent years, vacuum assisted excision, which is more cost effective compared to the BLES, has become the standard of care for this indication [34].

Second, the BLES procedure is a potential alternative for patients that are ineligible for surgical procedure or anesthesia, as it can be applied in an outpatient setting without general anesthesia. Nevertheless, complete excision of invasive lesions cannot be guaranteed with the BLES and such cancers can commonly be controlled with hormonal therapy alone [35].

Only one (9.1%) complication with the device was reported in this study, which is comparable with previous literature [9, 16, 23]. Another common complication due to the BLES system is bleeding (0–11.8%) and hematoma (0–8.8%) [9–18, 24, 25, 33, 36], none of which occurred during this study. This is probably because the conventional surgery was performed immediately after the BLES procedure.

The small number of patients (*n* = 22) is the major limitation of our study, especially considering that 10 patients (45%) were subsequently excluded due to MRI findings. However, it was the purpose of this study to select and evaluate only those patients with small lesions that had the best chance of a complete excision with the BLES device. In addition, during this study we did not assess the learning curve for our radiologists in the use of the BLES. However, the radiologists are experienced with different biopsy techniques under ultrasound guidance and all radiologists, surgeons and surgery assistants received a training program regarding the use of the BLES device. Furthermore, Michalopoulos et al. [37] stated that the BLES appears to be an easier-to-learn technique compared to the vacuum assisted biopsy (VAB) procedure, therefore it seems that any bias due to a lack of previous experience with this device is minimized.

In conclusion, based on previous studies and our own experience in selected cases, the BLES is a reliable diagnostic method with low underestimation and complication rates. However, we discourage its use for excision of malignant lesions as a substitute for surgical excision. In the future we hope that the design of the BLES will be modified and improved to make it better suitable for complete excision of small malignant lesions.

## Data Availability

The datasets generated during and/or analyzed during the current study are available from the corresponding author on reasonable request.

## References

[1] Verbeek ALM, Broeders MJM, Otto SJ, Fracheboud J, Otten JDMH, Holland R, Heeten GJAD, Koning HJD (2013). Effecten van het bevolkingsonderzoek naar borstkanker. Ned Tijdschr Geneeskd.

[2] Kaviani A, Sodagari N, Sheikhbahaei S, Eslami V, Hafezi-Nejad N, Safavi A, Noparast M, Fitoussi A (2013). From radical mastectomy to breast-conserving therapy and oncoplastic breast surgery: a narrative review comparing oncological result, cosmetic outcome, quality of life, and health economy. ISRN Oncol.

[3] Faverly DR, Hendriks JH, Holland R (2001). Breast carcinomas of limited extent: frequency, radiologic-pathologic characteristics, and surgical margin requirements. Cancer.

[4] Holland R, Veling SHJ, Mravunac M, Hendriks JHCL (1985). Histologic multifocality of tis, T1–2 breast carcinomas implications for clinical trials of breast-conserving surgery. Cancer.

[5] Fisher B, Jeong JH, Anderson S, Bryant J, Fisher ER, Wolmark N (2002). Twenty-five-year follow-up of a randomized trial comparing radical mastectomy, total mastectomy, and total mastectomy followed by irradiation. N Engl J Med.

[6] Darby S, McGale P, Correa C, Taylor C, Arriagada R, Clarke M, Cutter D, Davies C, Ewertz M, Godwin J, Gray R, Pierce L, Whelan T, Wang Y, Peto R, Early Breast Cancer Trialists' Collaborative G (2011). Effect of radiotherapy after breast-conserving surgery on 10-year recurrence and 15-year breast cancer death: meta-analysis of individual patient data for 10,801 women in 17 randomised trials. Lancet.

[7] Houssami N, Macaskill P, Marinovich ML, Morrow M (2014). The association of surgical margins and local recurrence in women with early-stage invasive breast cancer treated with breast-conserving therapy: a meta-analysis. Ann Surg Oncol.

[8] Sanderink WBG, Mann RM (2018). Advances in breast intervention: where are we now and where should we be?. Clin Radiol.

[9] Seror JY, Lesieur B, Scheuer-Niro B, Zerat L, Rouzier R, Uzan S (2012). Predictive factors for complete excision and underestimation of one-pass en bloc excision of non-palpable breast lesions with the Intact((R)) breast lesion excision system. Eur J Radiol.

[10] Sie A, Bryan DC, Gaines V, Killebrew LK, Kim CH, Morrison CC, Poller WR, Romilly AP, Schilling K, Sung JH (2006). Multicenter evaluation of the breast lesion excision system, a percutaneous, vacuum-assisted, intact-specimen breast biopsy device. Cancer.

[11] Allen SD, Nerurkar A, Della Rovere GU (2011). The breast lesion excision system (BLES): a novel technique in the diagnostic and therapeutic management of small indeterminate breast lesions?. Eur Radiol.

[12] Allen SD, Osin P, Nerurkar A (2014). The radiological excision of high risk and malignant lesions using the INTACT breast lesion excision system: a case series with an imaging follow up of at least 5 years. Eur J Surg Oncol.

[13] Diepstraten SC, Verkooijen HM, van Diest PJ, Veldhuis WB, Fernandez-Gallardo AM, Duvivier KM, Witkamp AJ, van Dalen T, Mali WP, van den Bosch MA (2011). Radiofrequency-assisted intact specimen biopsy of breast tumors: critical evaluation according to the IDEAL recommendations. Cancer Imaging.

[14] Medjhoul A, Canale S, Mathieu MC, Uzan C, Garbay JR, Dromain C, Balleyguier C (2013). Breast lesion excision sample (BLES biopsy) combining stereotactic biopsy and radiofrequency: is it a safe and accurate procedure in case of BIRADS 4 and 5 breast lesions?. Breast J.

[15] Razek NA, Eshak SE, el Ghazaly H, Omar OS, Yousef OZ, Shaalan M (2013). Percutaneous breast lesion excision system (BLES): a new tool for complete closed excision of high risk lesions (Egyptian experience). Egyptian J Radiol Nucl Med.

[16] Killebrew LK, Oneson RH (2006). Comparison of the diagnostic accuracy of a vacuum-assisted percutaneous intact specimen sampling device to a vacuum-assisted core needle sampling device for breast biopsy: initial experience. Breast J.

[17] Graham CL (2017). Evaluation of percutaneous vacuum assisted intact specimen breast biopsy device for ultrasound visualized breast lesions: upstage rates and long term follow-up for high risk lesions and DCIS. Breast.

[18] Niinikoski L, Hukkinen K, Leidenius MHK, Stahls A, Meretoja TJ (2018). Breast lesion excision system in the diagnosis and treatment of intraductal papillomas — a feasibility study. Eur J Surg Oncol.

[19] Eggemann H, Costa SD, Ignatov A (2016). Ultrasound-guided versus wire-guided breast-conserving surgery for nonpalpable breast cancer. Clin Breast Cancer.

[20] Ahmed M, Douek M (2013). Intra-operative ultrasound versus wire-guided localization in the surgical management of non-palpable breast cancers: systematic review and meta-analysis. Breast Cancer Res Treat.

[21] Dalmis MU, Litjens G, Holland K, Setio A, Mann R, Karssemeijer N, Gubern-Merida A (2017). Using deep learning to segment breast and fibroglandular tissue in MRI volumes. Med Phys.

[22] NABON: Richtlijn Mammacarcinoom versie 2.0, Breast Cancer Guideline. vol 2012.

[23] Scaperrotta G, Ferranti C, Capalbo E, Paolini B, Marchesini M, Suman L, Folini C, Mariani L, Panizza P (2016). Performance and role of the breast lesion excision system (BLES) in small clusters of suspicious microcalcifications. Eur J Radiol.

[24] Milos RI, Bernathova M, Baltzer PA, Pinker-Domenig K, Kapetas P, Rudas M, Helbich TH (2017). The breast lesion excision system (BLES) under stereotactic guidance cannot be used as a therapeutic tool in the excision of small areas of microcalcifications in the breast. Eur J Radiol.

[25] Papapanagiotou IK, Koulocheri D, Kalles V, Liakou P, Michalopoulos NV, Al-Harethee W, Georgiou G, Matiatou M, Nonni A, Pazaiti A, Theodoropoulos GE, Menenakos E, Zografos GC (2018). Margin-free excision of small solid breast carcinomas using the Intact Breast Lesion Excision System((R)): is it feasible?. Breast Cancer.

[26] Nasir N, Rainsbury RM (2003). The timing of surgery affects the detection of residual disease after wide local excision of breast carcinoma. Eur J Surg Oncol.

[27] Wiley EL, Diaz LK, Badve S, Morrow M (2003). Effect of time interval on residual disease in breast cancer. Am J Surg Pathol.

[28] Moran MS, Schnitt SJ, Giuliano AE, Harris JR, Khan SA, Horton J, Klimberg S, Chavez-MacGregor M, Freedman G, Houssami N, Johnson PL, Morrow M, Society of Surgical O American Society for Radiation O (2014). Society of Surgical Oncology-American Society for Radiation Oncology consensus guideline on margins for breast-conserving surgery with whole-breast irradiation in stages I and II invasive breast cancer. J Clin Oncol.

[29] Senkus E, Kyriakides S, Ohno S, Penault-Llorca F, Poortmans P, Rutgers E, Zackrisson S, Cardoso F, Committee EG (2015). Primary breast cancer: ESMO clinical practice guidelines for diagnosis, treatment and follow-up. Ann Oncol.

[30] Vos EL, Gaal J, Verhoef C, Brouwer K, van Deurzen CHM, Koppert LB (2017). Focally positive margins in breast conserving surgery: predictors, residual disease, and local recurrence. Eur J Surg Oncol.

[31] Vos EL, Siesling S, Baaijens MHA, Verhoef C, Jager A, Voogd AC, Koppert LB (2017). Omitting re-excision for focally positive margins after breast-conserving surgery does not impair disease-free and overall survival. Breast Cancer Res Treat.

[32] Christou A, Koutoulidis V, Koulocheri D, Panourgias E, Nonni A, Zografos CG, Zografos GC (2019). Performance of breast lesion excision system (BLES) in complete removal of papillomas presented mammographically as groups of calcifications. Clin Imaging.

[33] Sklair-Levy M, Rayman S, Yosepovich A, Zbar A, Goitein D, Zippel D (2018). The Intact((R)) breast lesion excision system as a therapeutic device for selected benign breast lesions. Breast J.

[34] National Institute for Health and Care Excellence (2006) Image-guided vacuum-assisted excision biopsy of benign breast lesions (interventional procedures guidance [IPG156]). https://www.nice.org.uk/guidance/ipg156. Accessed 06 April 2020

[35] Mustacchi G, Ceccherini R, Milani S, Pluchinotta A, De Matteis A, Maiorino L, Farris A, Scanni A, Sasso F, Italian Cooperative Group G (2003). Tamoxifen alone versus adjuvant tamoxifen for operable breast cancer of the elderly: long-term results of the phase III randomized controlled multicenter GRETA trial. Ann Oncol.

[36] Al-Harethee WA, Kalles V, Papapanagiotou I, Matiatou M, Georgiou G, Nonni A, Koulocheri D, Liakou P, Theodoropoulos G, Zografos GC (2015). Thermal damage of the specimen during breast biopsy with the use of the Breast Lesion Excision System: does it affect diagnosis?. Breast Cancer.

[37] Michalopoulos NV, Maniou I, Zografos GC (2012). Breast lesion excision system biopsy: the learning curve. AJR Am J Roentgenol.

